# Hymenoptera and biomimetic surfaces: insights and innovations

**DOI:** 10.3762/bjnano.15.107

**Published:** 2024-11-05

**Authors:** Vinicius Marques Lopez, Carlo Polidori, Rhainer Guillermo Ferreira

**Affiliations:** 1 Lestes Lab, Federal University of Triângulo Mineiro, Uberaba, Minas Gerais, Brazil; 2 Department of Environmental Science and Policy (ESP), University of Milan, Via Celoria 26, 20133, Milan, Italy

**Keywords:** arthropods, bio-inspired surfaces, bioengineering, cuticle, nanoscale structures

## Abstract

The extraordinary adaptations that Hymenoptera (sawflies, wasps, ants, and bees) exhibit on their body surfaces has long intrigued biologists. These adaptations, which enabled the immense success of these insects in a wide range of environments and habitats, include an amazing array of specialized structures facilitating attachment, penetration of substrates, production of sound, perception of volatiles, and delivery of venoms, among others. These morphological features offer valuable insights for biomimetic and bioinspired technological advancements. Here, we explore the biomimetic potential of hymenopteran body surfaces. We highlight recent advancements and outline potential strategic pathways, evaluating their current functions and applications while suggesting promising avenues for further investigations. By studying these fascinating and biologically diverse insects, researchers could develop innovative materials and devices that replicate the efficiency and functionality of insect body structures, driving progress in medical technology, robotics, environmental monitoring, and beyond.

## Introduction

The body surfaces of insects are marvels of natural engineering, displaying a remarkable array of adaptations that enable them to thrive in diverse environments [1–3]. Insects have developed a variety of mechanisms to cope with the challenges posed by their habitats, from specialized structures for attachment and movement to unique features that enhance survival and reproductive success [4]. These adaptations may provide valuable insights for biomimetic and bioinspired technological advancements [1]. Hence, understanding these mechanisms not only sheds light on the evolutionary ingenuity of insects but also offers innovative solutions and technological applications.

**Figure 1:**
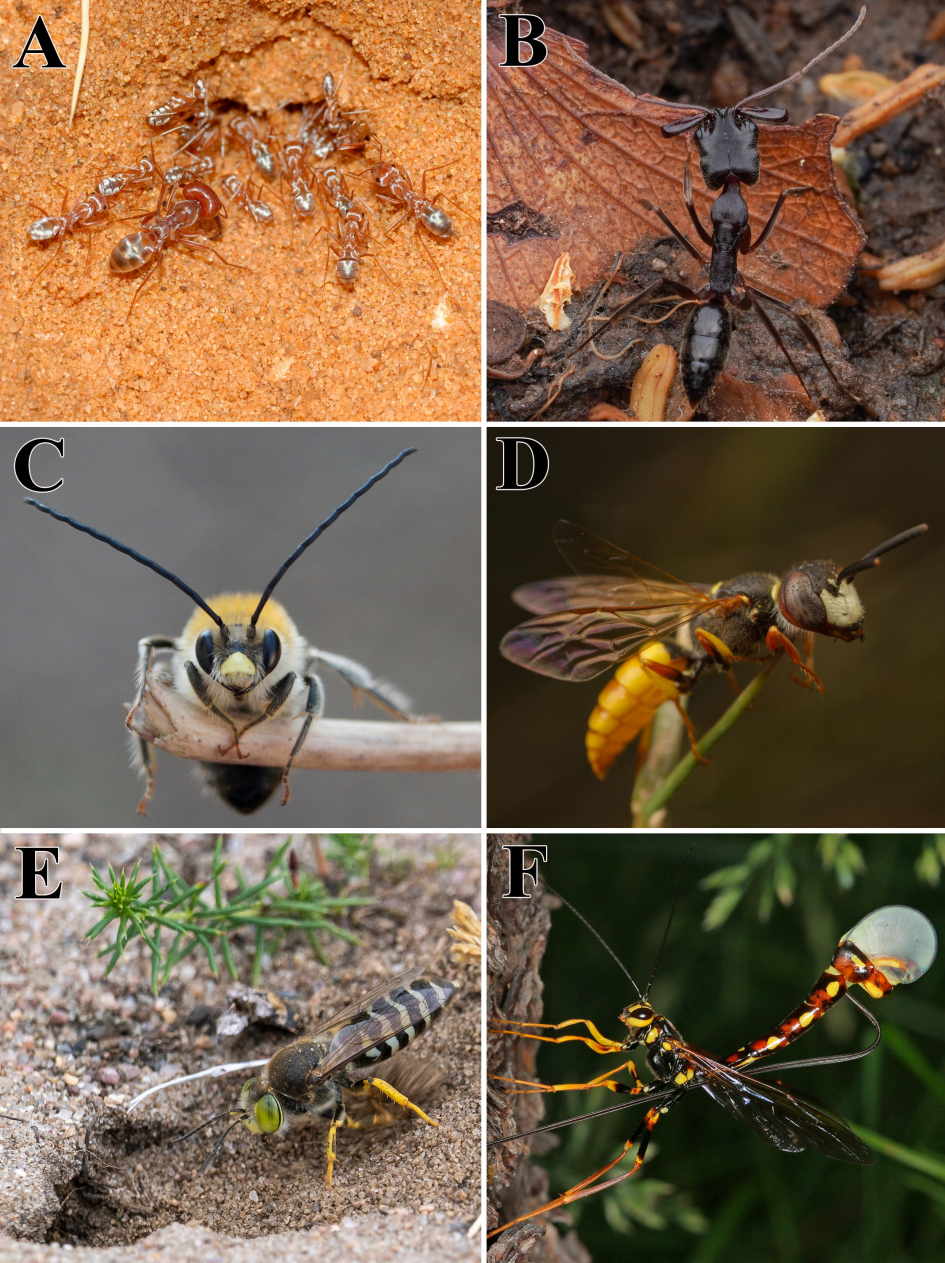
Diversity of hymenopteran species showcasing a range of shapes and life strategies. (A) Saharan silver ant (*Cataglyphis bombycine*) (© 2017 Manuel García-Viñó Sánchez). (B) Tap-jaw ants (*Odontomachus troglodytes*) (© 2024 Jonghyun Park). (C) Long-horned bees (*Eucera nigrescens*) (© 2020 Corinna Herr). (D) Beewolves (*Philanthus triangulum*) (© 2020 Johan Pretorius). (E) Sand wasps (*Bembix rostrata*) (© 2022 Piotr Lukasik). (F) Norton's giant ichneumonid wasp (*Megarhyssa nortoni*) (© 2015 Ed Oswalt). Figure A was taken by Manuel García-Viñó Sánchez and is used with permission. This content is not subject to CC BY 4.0. Figures B–F were reproduced from https://www.inaturalist.org/observations/204425768, https://www.inaturalist.org/observations/41894658, https://www.inaturalist.org/observations/45592397, https://www.inaturalist.org/observations/123874897 and https://www.inaturalist.org/observations/1778516 respectively (published by iNaturalist, distributed under the terms of the Creative Commons Attribution-Non Commercial 4.0 International License, https://creativecommons.org/licenses/by-nc/4.0/). This content is not subject to CC BY 4.0.

The order Hymenoptera, which includes sawflies, wasps, ants, and bees, is one of the most diverse groups in the class Insecta, with over 153,000 described species [5] and an estimated 1 million species yet to be discovered [6]. Traditionally, this order is divided into “Symphyta” (sawflies) and Apocrita. The primary distinction between sawflies and Apocrita lies in their body structure: Sawflies lack a “wasp waist” and exhibit a broad connection between the abdomen and thorax, similar to other insects. In contrast, Apocrita are characterized by the integration of the first abdominal tergite into the metathorax to form the propodeum. This results in a mesosoma that includes the thorax and propodeum, housing the primary locomotory structures, that is, legs, wings, and their musculature, which makes it the most complex skeletomuscular region of the insect. This structure is fundamental for generating flight power and precise wing adjustments. The remainder of the abdomen is known as the metasoma. The articulation between the mesosoma and metasoma, marked by the wasp waist (or petiole), enhances the maneuverability of the metasoma and its ovipositor or sting, allowing for efficient prey capture, defense, and oviposition (Figure 1).

Hymenoptera exhibit a remarkable variety of biological structures and functions, possessing highly specialized organs and body parts, each adapted to specific ecological roles and lifestyles. These morphological and functional innovations predominantly involve the slender waist of Apocrita wasps, the stinging mechanism observed in Aculeata, parasitoidism (a specialized form of carnivorous behavior), and secondary phytophagy (a reversion to plant-based feeding) [7]. Hymenoptera are also well known as the animal clade where complex forms of cooperative behavior (eusociality) arise most independent times, and such behavioral specialization also drove the evolution of novel morphologies related to, for instance, task specialization in the different castes [8–10]. Studying and emulating these features, scientists and engineers can develop innovative materials and devices that mirror the efficiency and functionality of Hymenopteran anatomy.

**Figure 2:**
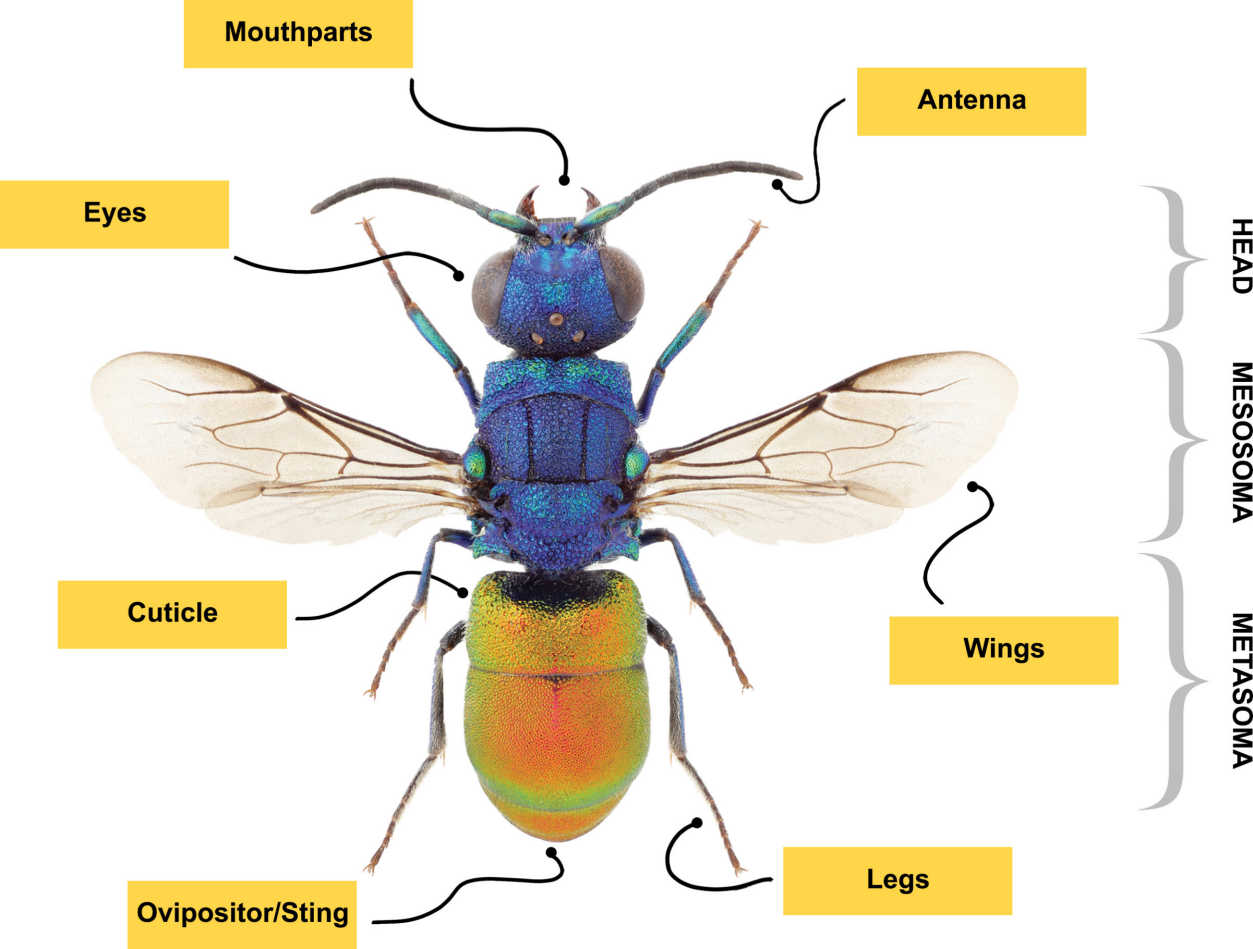
The body parts of Hymenoptera that can serve as sources for bioinspired and biomimetic materials and technologies. The image was adapted and reproduced from [11] (© 2015 J. Paukkunen et al., published by ZooKeys, distributed under the terms of the Creative Commons Attribution 4.0 International License, https://creativecommons.org/licenses/by/4.0).

Here we describe the structural adaptations on the surfaces of the body of Hymenoptera (Figure 2) with potential biomimetic applications. By analyzing their unique morphological features and the principles behind their functionality, we aim to identify key characteristics that can inspire innovative materials and technologies.

## Review

### General features of body cuticle

The cuticle of Hymenoptera exhibits several fascinating properties.

#### Cuticle roughness

The cuticle surfaces of some species reduce friction and wear, inspiring the development of low-friction materials and coatings. By mimicking these natural textures, it is possible to create synthetic materials that exhibit similar friction-reducing properties, leading to significant advancements in mechanical efficiency and durability. For instance, low-friction coatings inspired by Hymenoptera cuticles can be applied to materials to reduce wear and tear, thereby enhancing performance, and extending the lifespan of the material.

Surface roughness can have beneficial effects on the overall aerodynamic characteristics of artificial surfaces, such as rough coatings on high-speed trains [12], dimples on golf balls [13], and shark skin denticles on aircrafts [14]. Some micromachines can also benefit from micro- and nanostructures that create roughness on surfaces and influence aerodynamics and heat transfer [15]. The sculptured and thick cuticle of some hymenopterans is also associated with increased resistance to fractures and high pressures [16] and may also potentially reduce water loss [17–18]. Alternative hypotheses yet to be tested for the function of such complex cuticle sculpturing is the air drag reduction during flight, as many hymenopterans (e.g., chrysidids, scelionids, and mutillids) have dimples on the cuticle that might have the same aerodynamic effect as those on golf balls [13]. Setose cuticle in hymenopterans also exhibits an interesting function, namely, reducing the accumulation and deposition of particles [19].

Similarly, in the medical field, these coatings can be used on surgical instruments and prosthetics to minimize friction against biological tissues, reducing discomfort and improving the functionality of medical devices. Furthermore, in the field of electronics, low-friction surfaces can prevent the wear of moving parts in devices such as hard drives and printers, ensuring longer operational life and reliability. The mechanisms behind the cuticle’s friction-reducing properties also include the ability to repel dust and contaminants, which further extends the applications of these biomimetic materials. For instance, the cuticle of sawfly larvae has complex nanostructures and wax crystals that result in hydrophobicity [20]. By incorporating these properties, manufacturers may develop self-cleaning surfaces that maintain their low-friction characteristics even in harsh environments. This feature is particularly valuable in aerospace applications, where equipment is often exposed to extreme conditions.

#### Coloration

Cuticle coloration in Hymenoptera may be achieved through either structural coloration or pigments, the latter being essentially melanin (eumelanin and/or pheomelanin) [21–23] (but see [24–25] for other pigments). Especially structural coloration provides a wealth of inspiration for creating vivid, durable colors without the use of dyes. This natural phenomenon involves the manipulation of light by micro- and nanostructures on the cuticle (e.g., epicuticular multilayer reflectors), which can produce brilliant and iridescent colors [26–28]. The examples range from the metallic wings of some bees to the striking iridescent colors of wasps [29–30]. Blue coloration is hard to find in nature because blue pigments are rare; hence, animals tend to evolve structural coloration that reaches blue hues, like in carpenter bees [31]. Some species have ultrablack cuticles that absorb nearly all incident light (Lopez et al., this volume), a feature that can be used to create highly efficient light-absorbing materials for solar panels [32–33]. Understanding and mimicking these biological systems can lead to significant advancements in various fields, including materials science, fashion, and environmental sustainability [34].

In materials science, the principles of structural coloration can be applied to develop colorfast materials that do not fade over time [34–37]. Traditional pigments and dyes can degrade under exposure to light, heat, and chemicals, but structurally colored materials maintain their vibrancy indefinitely. This has potential applications in creating long-lasting paints, coatings, and fabrics, reducing the need for frequent replacements and repainting, thus conserving resources, and reducing waste [34]. The fashion industry can also benefit greatly from such biomimetic and bioinspired surfaces [38]. By incorporating structural coloration into textiles, designers can create clothing and accessories with striking, iridescent hues that do not rely on chemical dyes [38]. This can lead to more sustainable fashion practices, as the production of synthetic dyes often involves toxic chemicals and generates significant environmental pollution [38]. Structurally colored fabrics would offer a greener alternative, aligning with the growing demand for eco-friendly fashion.

Furthermore, structural coloration can inspire the creation of innovative security features [34]. The unique optical effects of these natural structures are difficult to replicate and counterfeit, making them ideal for use in anti-counterfeiting measures. Banknotes, identification cards, and high-value documents can incorporate structural coloration to enhance security and prevent forgery, leveraging the complexity and uniqueness of these bioinspired designs. In the field of consumer electronics, the vibrant and durable colors produced by structural coloration can be used to create more aesthetically pleasing and durable electronic devices. Smartphone cases, laptops, and other gadgets can feature iridescent colors that do not wear off or fade, enhancing both their appearance and longevity. This not only improves the user experience but also contributes to a reduction in electronic waste, as devices retain their visual appeal over a longer period. The techniques to create biomimetic materials with color producing mechanisms inspired by insects already exist [29–30] and applications of such technology have several possibilities.

#### Hairs

The body surface of insects is equipped with hairs (sensu lato) with different morphologies. These structures may first be categorized into two main types, that is, setae, which have a socket (which originates from an adjacent cell) and microtrichia (not socked and thus originating from one cell) [39–40]. Such tiny structures belong to two main functional types. They are either mechanosensory and belong to the peripheral nervous system or they have no sensing role and serve to prevent wetting of, for example, wings and legs [41–44]. Other functions include the detection of airflow patterns, for example, through trichoid sensilla on the compound eyes of honeybees [45], which are important to maintain and coordinate flight. In general, the head capsule of Hymenoptera is densely covered with hairs and may be a ground-plan feature of Hymenoptera and a potential autapomorphy [46]. While the reason for such trait is still unknown, the fact that another order, the Diptera, also including mostly quick-flying insects, show the same pattern, may suggest a relationship with optimization of flight behavior. Evidence on structures and functions of some of these non-sensory hairs in ants and bees suggest intriguing applications in biomimetics.

In ant larvae, other non-sensory functions of hairs include, for example, ensuring ventilation at the body surface [47] and larval clumping, the latter function through special “hooked” hairs [48]. Adult ants of the tropical tribes Basicerotini and Stegomyrmecini, in contrast, possess brush hairs on the body that capture minute soil particles, camouflaging themselves with the soil surface [10], while adult desert ants have special long and curved hairs on the lower surface of the head and mouthparts, which improve soil digging and soil carrying [49].

In the honeybee, a special microscale hairy compliant texture on abdominal surfaces reduces friction, which is relevant considering that the abdominal sections, by undergoing many reciprocating motions, are at risk of wear or abrasion [50]. Ocular hairs in honeybees reduce airflow at the eye surface by up to 90%, deflecting incoming air and create a zone of stagnant air, potentially helping in the deployment of sensors outdoors, where they are strongly subjected to airborne dust [19].

The detailed study of hymenopteran hairs can be useful in fabricating new surfaces with friction reduction mechanisms and, consequently, longer life time. Special hairs that may be useful to create surfaces optimized for protection from overheating are those responsible for the silver appearance of the Saharan silver ant *Cataglyphis bombycine* [51] (Figure 1A). These hairs have a particular, rarely found morphology (triangular cross section with two corrugated surfaces) associated with a strong optical reflection in the visible and near-infrared (NIR) range, while maximizing heat emissivity in the mid-infrared (MIR). This allows the insects to maintain a lower thermal steady state and to cope with high temperatures (50 °C or even more) [51]. Additionally, specialized hairs with thermal functions are present in both wasps [52] and bees [53].

At last, hymenopteran hairs considerably vary in terms of length [54–56], suggesting that they may possess different physical properties, which may be variably used in biomimetic applications.

### Specialized structures on the three-body tagma

#### Head

**Figure 3:**
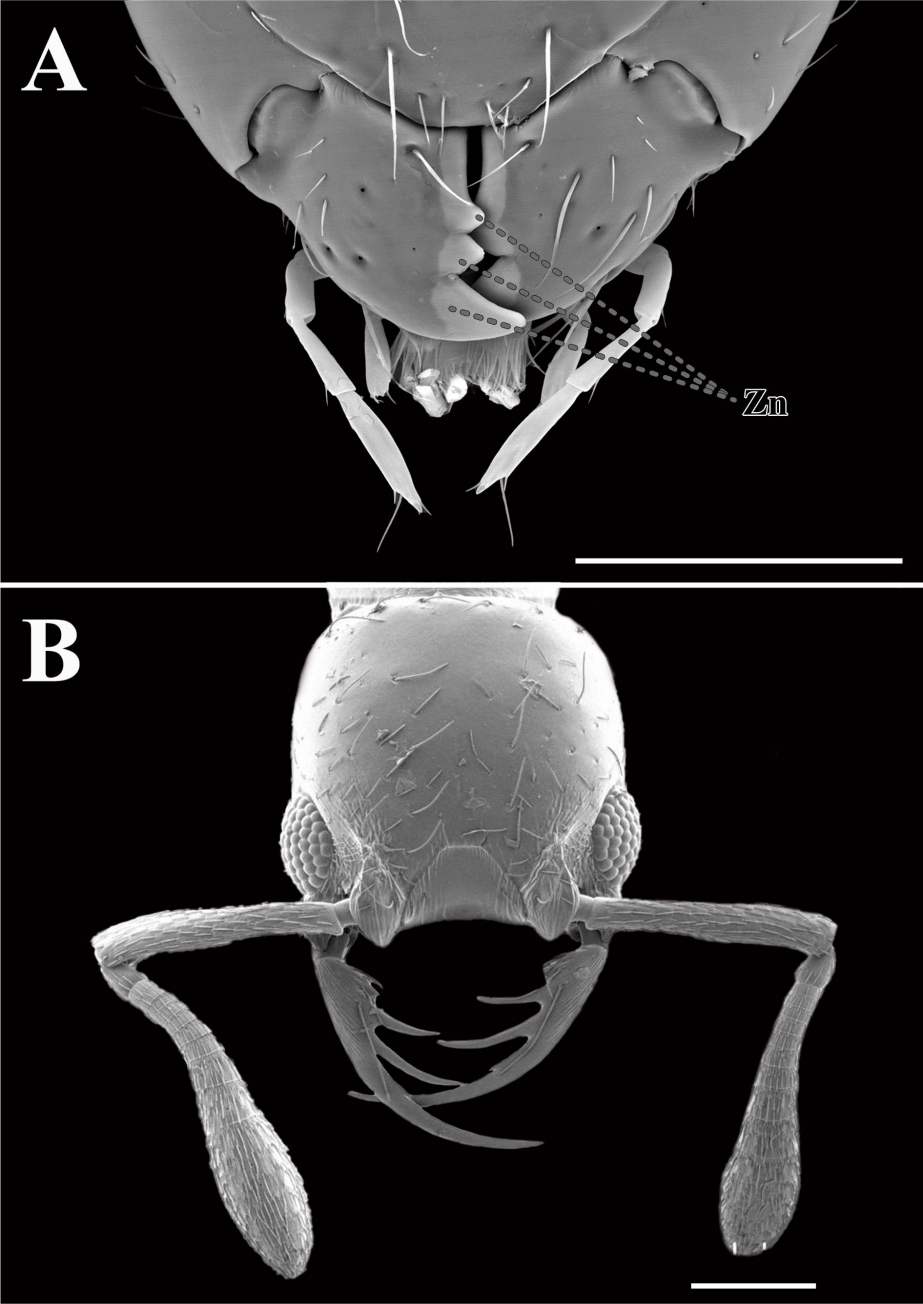
Scanning electron microscopy (SEM) images of ant mandibles. (A) Zinc-enriched mandibles of *Aganaspis daci*. (B) Jaw of *Thaumatomyrmex fraxini*. Zn: Zinc. Scale bars: 200 µm. Figure B was adapted from https://www.antweb.org/bigPicture.do?name=antweb1008597&shot=h&number=2 (© 2024 California Academy of Sciences, uploaded by R. Keller, specimen code ANTWEB1008597, published in AntWeb Version 8.108, accessed 13 August 2024, distributed under the terms of the Creative Commons Attribution 4.0 International License, https://creativecommons.org/licenses/by/4.0).

**Mouthparts:** The mouthparts of Hymenoptera, which are adapted for a variety of functions such as cutting, chewing, and sucking, provide valuable models for designing versatile tools and instruments. Within certain lineages of Hymenoptera, mandibles are known to include trace metals, mainly Zn and secondarily Mn, in the cuticle [57]; these features increase their hardness [58] (Figure 3A). Ants are by far the hymenopteran group where this trait was most extensively studied (21 species in total spanning five subfamilies), with the leaf-cutter genus *Atta* overrepresented in cuticular composition investigations (reviewed in [59]). Within Hymenoptera, ants are particularly fascinating because of their division into castes with distinct tasks and sizes, showcasing a hierarchical organization in their societies [60]. Among these castes, the mandibles play a critical role, with different sizes and functions correlating to specific tasks within the colony [60]. Studies found that larger leafcutter ants (*Atta laevigata*, Attini) have higher Zn content in their mandible cutting edges, leading to greater hardness and Young’s modulus [61]. Understanding the mechanical properties and composition of leafcutter ant mandibles could offer valuable insights into biomimetic design principles, potentially inspiring the development of innovative tools and instruments with enhanced performance and adaptability for various applications [62–63]. Recently, the mandibles of leafcutter ants have inspired the design of a biomimetic stubble-cutting device, showcasing excellent performance and potential applications in agriculture [64].

Ant mandibles offer valuable inspiration for the enhancement of medical tools and devices. For instance, bioabsorbable surgical clamps modeled after the morphology and topography of the *A. laevigata* mandible, characterized by smooth internal regions and rougher external surfaces, could significantly improve grip and functionality [65]. Furthermore, the unique kinematic features of ant mandibles, such as the mobile joint axis and the tilt in the mandibular axis, provide insights for designing more efficient gripping devices [66]. Recently, a commercially available endoscopic needle holder was developed based on the morphology of *Formica rufa*, resulting in a remarkable increase in force amplification by up to 296%, with experimental measurements showing an increase of up to 433%, without altering the tool’s size [66].

Mandibles can offer structural adaptations to deliver powerful and high-speed strikes, as in trap-jaw ants (e.g., *Odontomachus monticola*) [67] (Figure 1B). In these species, hollow mandibles combined with resilient fibrous helical structures enhance energy absorption and improve stress redistribution, providing additional protection against damage caused by impact loads [68]. Such adaptations not only facilitate efficient prey capture but contribute to the overall durability of the mandibles. Additionally, when threatened, these ants possess the remarkable ability to jump several centimeters propelled by the force of their mandibles [69]. Engineers and material scientists can draw inspiration from these natural designs to develop lightweight yet durable components that enhance energy absorption and mitigate damage from impact loads, thereby improving the safety and longevity of vehicles and aircraft.

The double-rowed teeth of primitive asian jumping ant (*Harpegnathos venator*), which confer enhanced tribological stability, provide a more stable coefficient of friction when pinching objects of varying sizes using different regions of the mandible [70]. This ingenious natural design could inspire the development of multifunctional robotic grippers, offering improved stability and adaptability in handling diverse objects [70]. The specialized mandibular morphology and task-specific bite mechanics observed in big-headed ants (*Pheidole*) offer insights into creating robust and efficient cutting tools that can perform specialized tasks with precision [71]. Other species awaiting study may possess even greater potential for fine object manipulation. For example, *Probolomyrmex* and *Thaumatomyrmex* (Figure 3B), which employ their specialized mandibles to capture Polyxenid millipedes and then strip them of their detachable and hazardous bristles [72–73]. Therefore, these biological inspirations can lead to the creation of advanced devices with improved performance and multifunctionality, pushing the boundaries of current engineering.

Honeybees must visit approximately 3000 flowers to produce a single gram of honey [74]. To accomplish this, they use their hairy tongues to dip into viscous nectar at high frequencies. Their mouthparts consist of a pair of galeae, labial palps, and a hairy glossa with a flabellum at the tip [75–76] (for a more detailed view of the mouthparts, see [77]). The different mouthparts combined with the characteristics of viscous food can inform the design of efficient viscous micropumps [78]. For instance, the galea ridges on the mouthparts of an Italian honeybee (*Apis mellifera ligustica*) facilitate nectar-dipping by minimizing drag, enabling the bees to feed more efficiently [75]. The unique morphology and dynamic movement of the bee’s hairy tongue optimizes nectar feeding while conserving energy, providing insights into design methodologies for fluid transport devices using hairy beds [79]. Even with a damaged tongue, bees can feed normally, indicating the presence of compensatory mechanisms [74,78]. For instance, increasing the dipping frequency and utilizing the hairs can offset nectar loss caused by a damaged tongue, which can be valuable in engineering applications [78,80]. Additionally, the interaction between bee mandibles and propolis highlights the potential for developing anti-adhesive surfaces [81]. Bioinspired surfaces based on honeybee mandibles have been shown to reduce propolis adhesion by over 40% compared to control surfaces, demonstrating significant potential for application in various industries [82].

**Eyes:** Similar to insects and other arthropods, hymenopterans possess compound eyes, consisting of numerous small visual units called ommatidia. Each ommatidium acts as an individual photoreceptive unit, collectively providing a panoramic view that offers several visual advantages, such large field of view, high temporal resolution, rapid capture and tracking of fast-moving objects [83–84]. This renders compound eyes particularly suitable for electronic surveillance applications, where broad observation coverage is essential for detecting multiple objects simultaneously [85–86]. They also hold potential for endoscopic examination [87] and robot navigation [88]. Recently, an innovative microfluidic-assisted 3D printing technique has facilitated the creation of a compound eye inspired by eyes of worker bees [89]. This innovative perspective can pave the way for various applications, ranging from advancements in endoscopic imaging to improvements in machine vision, ultimately enhancing the visual efficiency of robots and sensors in autonomous vehicles [89–91].

**Figure 4:**
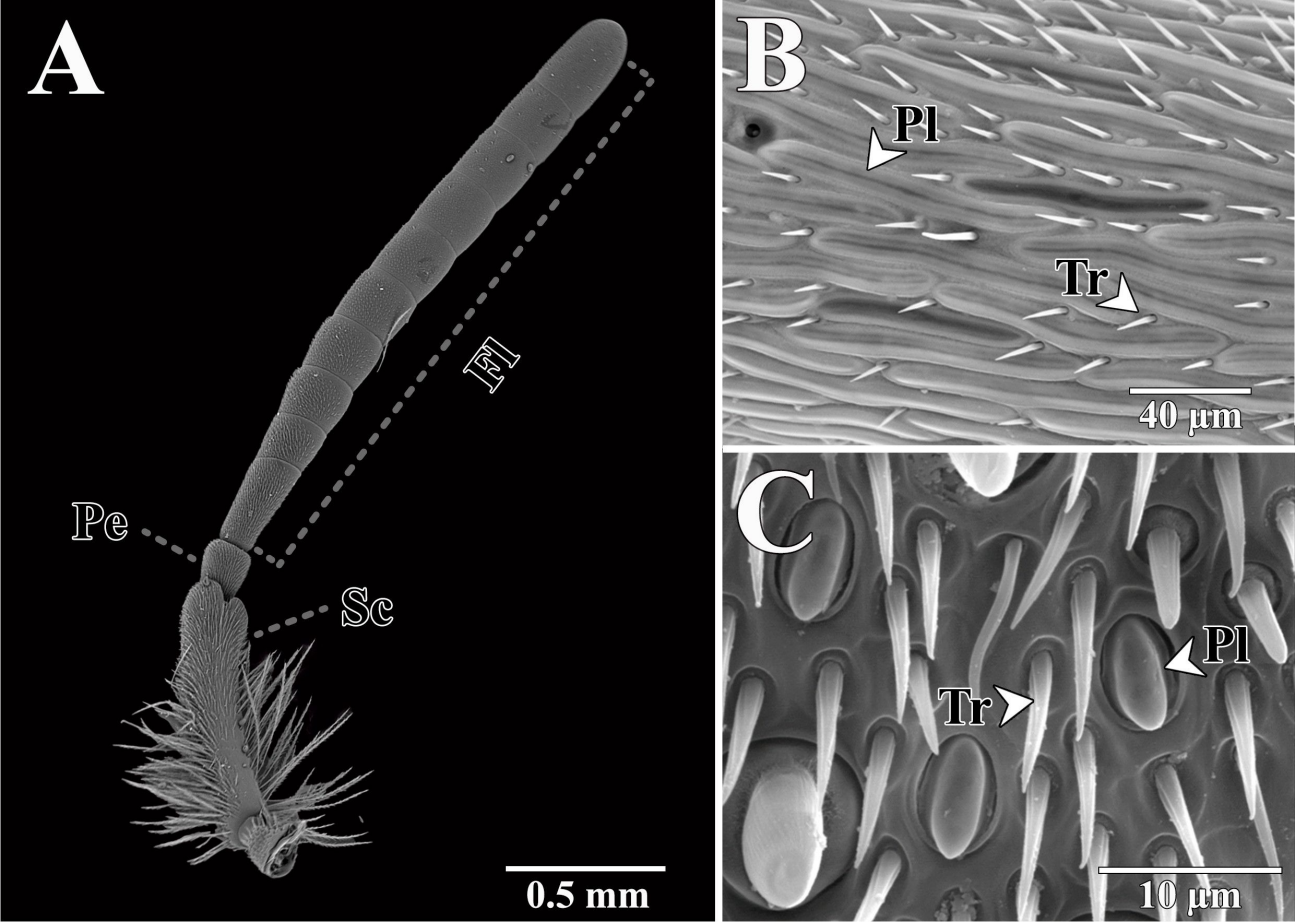
(A) SEM image of a hymenopteran antenna (*Anthidium oblongatum*). (B) SEM images of male antennal sensilla of *Ibalia leucospoides*. (C) SEM images of female antennal sensilla of Cerceris rubida. Pe: pedicel; Sc: scape; Fl: flagellum; Pl: placoid sensilla; Tr: trichoid sensilla.

**Antenna:** Hymenopteran antennae are equipped with specialized structures for detecting chemical, mechanical, and hygrothermal/CO_2_ cues in the environment, overall known with the term sensilla (Figure 4). Hymenoptera sensilla encompass different morphologies and sizes, which also vary in number both among species and between sexes [54–55]. Such great variability is often the result of co-evolution of these traits with ecological requirements [56,92–94]. Furthermore, they are essentially in both intra- and interspecific communications [95–97]. These antennal sensors have inspired the development of devices for the detection of volatile compounds, which have applications in environmental monitoring, food safety, and medical diagnostics [98]. The mobility of the antennae of long-horned bees (e.g., *Eucera longicornis*) are used by males to court females by gently grasping and pressing their antennae (Figure 1C). The antennae of long-horned bees and other hymenopterans may give some insight into microfilaments to grab small and delicate objects [99].

Female European beewolves (*Philanthus triangulum*) (Figure 1D) and other crabronids have developed a remarkable symbiotic relationship with bacteria of the genus *Streptomyces*, which they cultivate in specialized antennal glands [100–101]. This association is unique in that the bacteria are grown in large reservoirs within the antennae, where they receive nutrients from the gland cells. When the beewolf constructs its subterranean brood cells, it secretes these bacteria into the cells, where they produce antibiotic substances that protect the developing larvae from fungal infections. The antennal glands are highly specialized, featuring complex morphology including a monolayered epithelium and numerous gland units that facilitate the cultivation and secretion of the symbiotic bacteria. This intricate system ensures the survival of the offspring by creating a microenvironment hostile to pathogens, showcasing a fascinating example of insect–microbe symbiosis.

The ability of these wasps to maintain and apply symbiotic bacteria through their antennal glands can inspire the development of bioactive medical devices. These devices can release antimicrobial agents or probiotics to prevent infections and promote healing. For example, catheters and implants can mimic the wasp structures to reduce the risk of infection and enhance the body’s natural healing processes.

#### Mesosoma

**Wings:** In order to fulfill their essential functions, insect wings must effectively transmit force from the muscles at their base to the surrounding air, generate lift, and uphold structural integrity without deformation [102]. Hence, wings need to be lightweight, flexible, and resilient, rendering them captivating subjects for biomimetic materials research [102–103]. For instance, an examination of the nanomechanical properties of membranous wings in Chinese bees (*Apis cerana cerana*) reveals a synergistic interplay between veins and membranes, facilitating efficient load transmission and resulting in exceptional mechanical performance and structural stiffness [104]. Research on biological membranes, inspired by the superior structures of insect wings, holds potential for advancements in various sectors, including medical, construction, aviation, and automotive industries [104–108].

Research on hymenopteran wings remains limited, with many groups still requiring fundamental studies on their properties. Exploring other Hymenoptera species could yield valuable insights. For instance, the females of scoliid wasps, solitary parasitoids of scarab beetle larvae, dig into the ground to locate these larvae. This could inspire the development of wings for exploration and rescue drones, modeled after the scoliid wasps’ wings, offering high maneuverability, durability, and efficiency in flight and underground across varied terrains. Similarly, Vespidae with elongated bodies possess pointed wings for enhanced maneuverability, while stouter species have rounded wings for potentially higher flight speeds [109]. This variation could inform the design of modular drones capable of altering wing shapes to optimize either maneuverability or flight speed as needed. Additionally, the design of these devices could be enhanced by a unique feature observed in ensign wasps (*Afrevania* and *Trissevania*, Evaniidae) [110]. These wasps exhibit a sophisticated ability to fold their forewings along two intersecting fold lines, creating a four-plane wing folding mechanism [110]. By incorporating a similar four-plane wing folding mechanism, drones could achieve enhanced compactness and versatility, allowing for easy transport and storage, as well as efficient adaptation to various environments and mission requirements.

**Figure 5:**
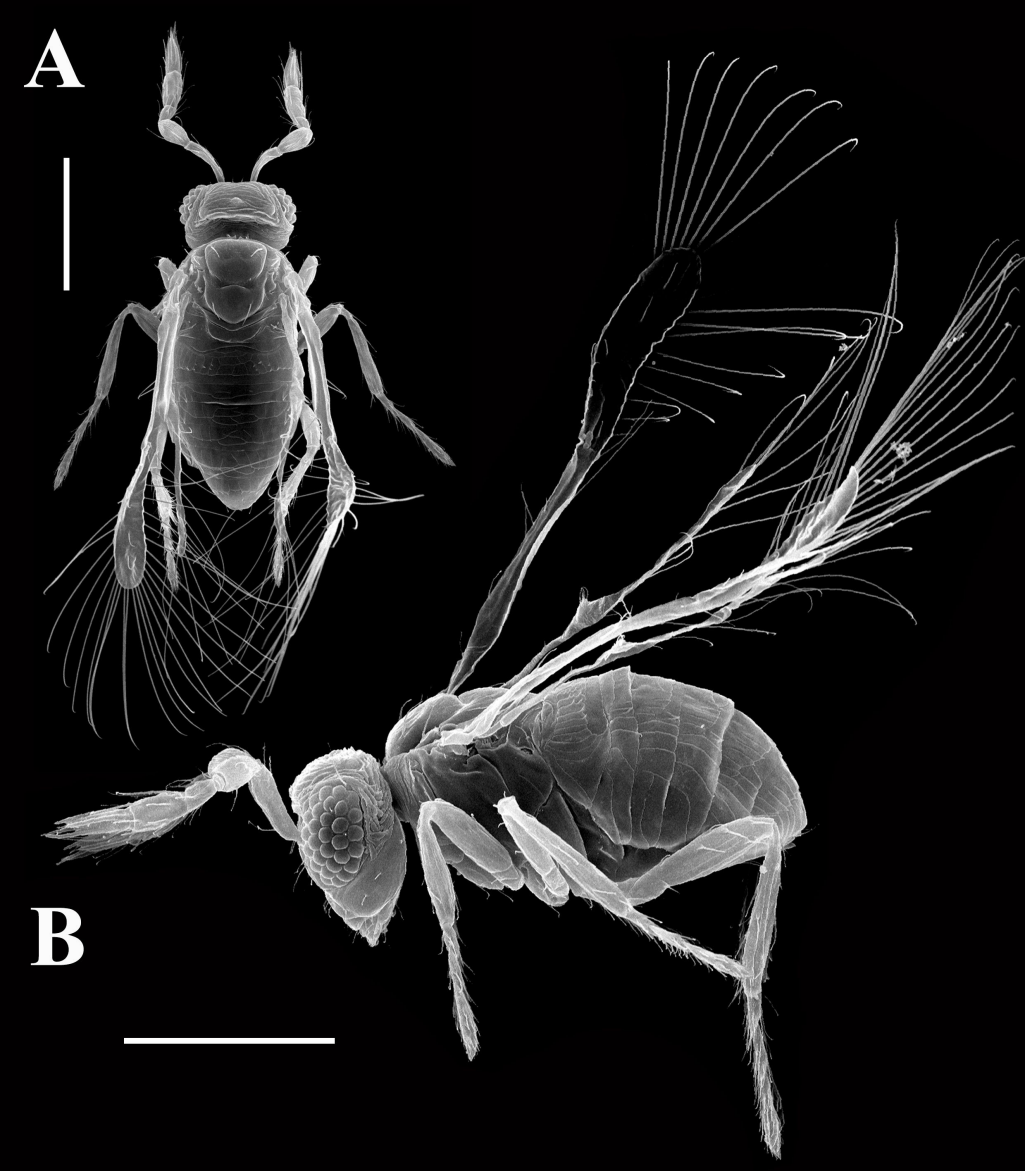
SEM image of wing bristles in a microwasp *Megaphragma polilovi*. (A) Dorsal view and (B) lateral view. Scale bars: 100 µm. Both figures are from [120] and were reprinted by permission from Springer Nature from the book “At the Size Limit - Effects of Miniaturization in Insects” (chapter “Structure of the Principal Groups of Microinsects. VI. Trichogrammatid Wasps (Hymenoptera: Trichogrammatidae)” by A. A. Polilov), Copyright 2016 Springer International Publishing Switzerland. This content is not subject to CC BY 4.0.

Microwasps exhibit remarkable adaptations in their wing structure, offering unique insights for biomimetic applications. Many families of microwasps are tiny, with adults measuring less than 2 mm, and their wings exhibit a distinctive morphology (Figure 5). For instance, in many microwasps (e.g., fairyflies (Mymaridae)), wings are predominantly composed of long bristles, with diameters ranging from 300 nm to 2.5 μm [111]. Although the functional basis of this morphology is not fully understood, these bristle-based wings may enable microwasps to sustain prolonged flight without the energetic costs typically associated with muscle activity [112–114]. Similar results are observed in the tiny beetle *Paratuposa placentis* (body length 395 μm), where the reduced wing mass and specific wing movement patterns contribute to enhanced flight performance [115]. In the context of biomimetic applications, understanding and replicating these adaptations can lead to significant advancements of micro-aerial vehicles [103]. By mimicking the lightweight, bristle-based wing structures of microwasps, engineers can develop microdevices that require less power to operate, thus extending their flight times and improving energy efficiency [116–117]. However, microwasps with other wing morphologies can also offer valuable insights and applications for the development of micro-aerial vehicles [118–119].

The wing-to-wing coupling mechanism in Hymenoptera functions as a multifaceted joint, linking the forewing’s rolled membrane to the hindwing’s hook structures, enabling synchronized movement and improved aerodynamic performance [121–122]. This mechanism is composed of a rolled membrane positioned at the trailing edge of the forewing, accompanied by small hooks (or hamuli) arranged in a line along the leading edge of the hind wing, all attached to a vein at the leading edge of the hind wing where the hooks are embedded [123]. These hooks are movable and exhibit an elastic base to ensure the high mobility of wings [124]. The coordinated movement of wings facilitated by this mechanism enables synchronized action and improved aerodynamic performance, while also allowing for decoupling during periods of rest, thereby avoiding aerodynamic interference and ensuring optimal flight dynamics. Computational models also indicate that the wing-to-wing coupling mechanism in Hymenoptera results in increased lift and drag, with the drag experiencing a higher rate of increase [122].

Despite their seemingly delicate nature and occupancy of a mere ≈0.2% of the total wing area, these hooks play a crucial role in continuously transferring forces between the wings, withstanding forces up to 180 times the insect’s body weight and 40 times the aerodynamic forces encountered during flight [123]. This robust design of the coupling mechanism is essential for maintaining functionality, particularly in scenarios involving frequent collisions, where it must endure forces surpassing typical flight stresses [123]. Additionally, the microstructural properties of the wings contribute to their hydrophobicity and anti-fouling capabilities, which can be applied to the development of self-cleaning surfaces and materials resistant to biofouling in marine environments.

#### Legs

**Figure 6:**
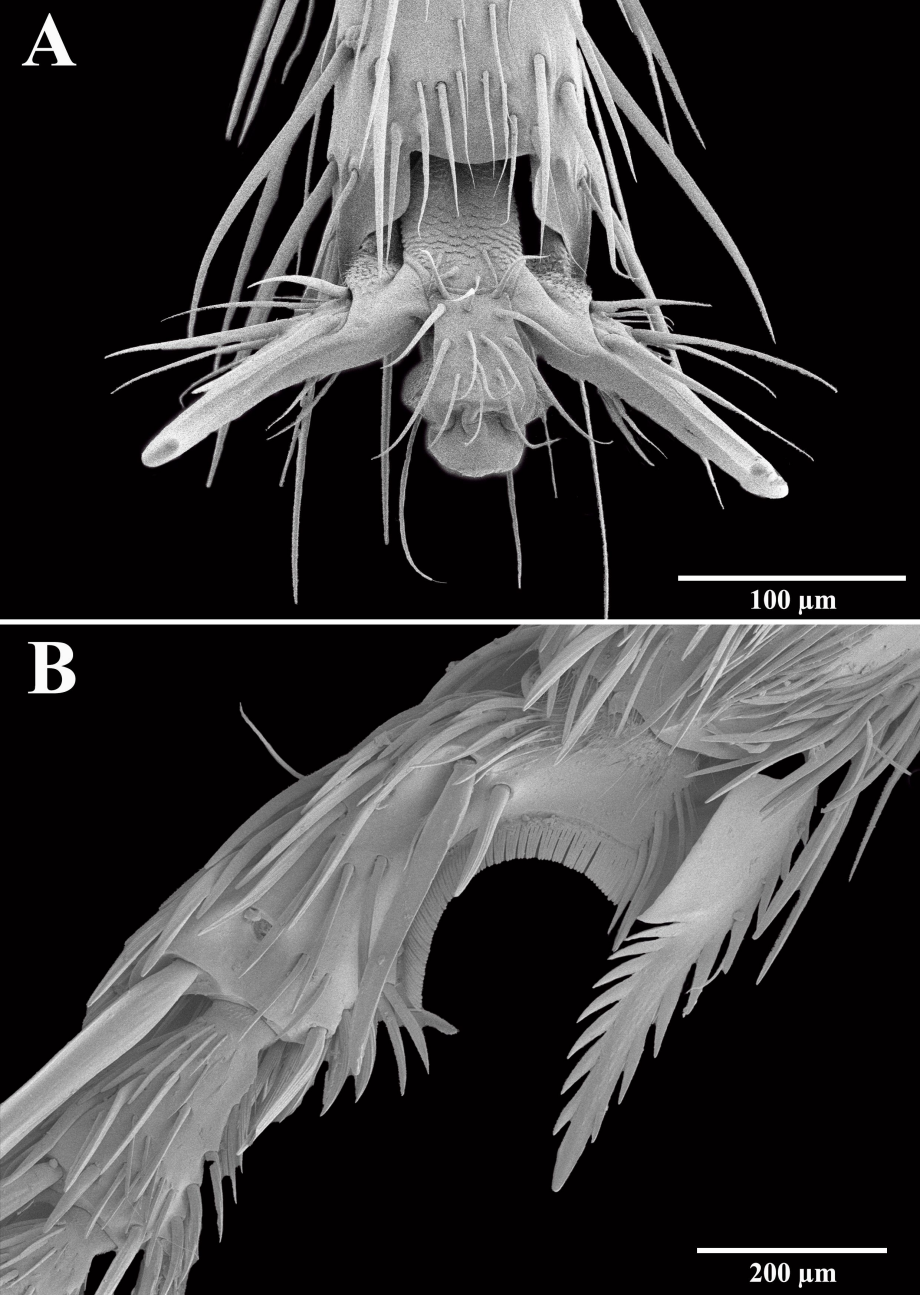
Structures on the leg of Hymenoptera. (A) Adhesive pads in ants (*Messor wasmanni*) and (B) antenna cleaner (strigil) of velvet ants (*Traumatomutilla bifurca*). Both figures were taken by Stanislav N. Gorb and were used with permission. This content is not subject to CC BY 4.0.

**Adhesive pads:** The adhesive organ in Hymenoptera consists of a flexible cuticle pad (i.e., arolium) situated between the pretarsal claws, capable of unfolding and retracting with each step [125] (Figure 6A). The arolium may be structured in lines perpendicular to the longitudinal axis of the pretarsus [126]. When extended (actively or passively) [127], the arolium comes into contact with the surface, thereby enhancing its adhesive contact area. Hymenopteran species serve as valuable sources of inspiration for artificial adhesive surfaces because of their rapid stepping frequencies [127]. For instance, weaver ants (*Oecophylla smaragdin*a) can swiftly adjust and control their contact areas in less than a millisecond, a capability that helps prevent unexpected detachment and enables efficient locomotion with a smaller contact area [128]. In honeybees, these pads function in response to specific drag activities, even without neuromuscular reflexes [129]. This passive mechanism is attributed to the structural characteristics of the soft pads, which work in concert with hierarchical structures supported by numerous branched internal fibers [129]. Moreover, the pads in Hymenoptera exhibit self-cleaning capabilities [130]. The precise control of adhesive strength and contact area can inspire the development of new bioinspired surfaces that significantly reduce switching times between attachment and detachment.

In other instances, the presence of curved spines or hair on the tarsomeres enhances locomotion on irregular surfaces by penetrating the microdevices of the substrate, providing thousands of interlocking points that contribute to overall friction [131]. For example, weaver ants, renowned for their ability to cling to vertical surfaces and construct large leaf nests, utilize dense arrays of tarsal friction hairs on their tarsomeres to boost adhesion on heavily sculpted surfaces [132–133]. Similarly, females of *Anastatus bifasciatus* (Eupelmidae) employ these structures to achieve a firm grip on the uneven surfaces of host eggs [134]. These examples highlight the potential for biomimetic applications of such structures in developing advanced adhesive technologies and improving robotic mobility on uneven terrain and in microgravity environments [135].

**Corbicula:** Several bee species (e.g., honey bees, bumble bees, and orchid bees) exhibit a pollen basket on the hindlegs called corbicula [136]. Bees pack pollen grains along with vegetal resins and nectar in their corbiculae and take flight with this material without losing the attachment force even under great air drag and movement-derived forces [136]. Corbiculate bees are one of the most effective pollinator groups worldwide. Up to now, there are no studies on the potential replication of such strategy in biomimetics; however, one might suggest that the successful evolutionary history of the corbicula may be a source of inspiration for technology. Potentially, the corbicula, as other structures mentioned here, may inspire the creation of materials and innovations to carry viscous substances across large distances.

**Micro-grooming tools:** The maintenance of clean and functional body surfaces is crucial for insects. The typical mode of grooming occurring in Hymenoptera consists in scraping (one-directional movement of one structure against another), rubbing (two-directional movement of one or both structures in contact) or nibbling (an antenna or leg is taken into the inner mouthparts, essentially mandibles or legs) [137]. About 30 distinct types of grooming movements are actually recognized for the order [138].

The micro-grooming tools of Hymenoptera, used for cleaning and maintaining their bodies, provide models for the development of micromanipulation and cleaning devices in nanotechnology and microsurgery. This is especially true for the specialized two-part cleaning structure on their front legs used to clean their antennae efficiently [139]. This antenna cleaner (strigil) (Figure 6B) consists of an apical and modified protibial spur (calcar, composed of a trunk and a velum) and a modified basitarsus including a fine comb made up of setae and a notched inner surface. This structure was observed to be morphologically highly variable even within hymenopteran families [140], which may suggest that different habitat features (e.g., forests vs open grasslands or sandy soils vs clay soils) may require highly adapted cleaning structures. Simulating the cleaning strokes of the tarsal notch and tibial spur on contaminated antennae in *Camponotus rufifemur* ants demonstrated that both components effectively removed particles [141]. The cleaning occurs through both macroscopic contact and microscopic interactions between the cleaning hairs, antennal sensilla, and contaminating particles. Microscopic combs and brushes act as filters for particles of different sizes, with larger particles being scraped off by the bristles and comb, while smaller particles are picked up by the brush’s flexible setae.

In the honeybee, hairs on the legs serve not only as an adhesive structure for pollen, but also for pollen removal. This is necessary since otherwise pollen widely distributed on the body would make sensing and controlled flight difficult. In particular, hair spacing and geometry on the forelegs affects the ability of pollen removal from the eyes (which are also equipped with adequately spaced hairs to promote their cleaning) [142]. All these findings highlight the sophisticated design of Hymenopteran cleaning structures, which can inspire synthetic cleaning technologies at the micro- and nanoscales, potentially improving the fabrication of delicate devices by reducing contamination-induced defects.

**Digging:** Many species of bees, wasps, and ants nest in the ground, showing a remarkable digging ability [143] (Figure 1E). Digging can be very efficient even in very hard soils [144], with some species excavating tunnels up to 1 m or more below the surface [145]. This activity is possible through the use of legs and mouthparts with morphologies highly adapted to this task, including, for example, robust spurs (legs) and large mandibles [146–148]. Hence, these structures and their movements while digging may be used as bionic prototypes for the design of low-resistance soil-engaging components, similar to the approach previously utilized with other insects as design models (e.g., mole crickets) [149].

Also interestingly, different Hymenoptera species have strong preferences for different types of soil [150]. Some bee and wasp species nest in highly sandy soils, while other species nest in very hard and silt- or clay-rich soil. Different soils, in turn, require different strategies of efficient digging, which can inspire different artificial instruments specialized in each soil type. This was already done using other insects as models, such as antlions, which dig in largely sandy soils and whose excavating behavior and related morphological structures inspired a biomimetic subsoiler tip that can reduce draught force in such type of soil [151–152].

#### Metasoma

**Figure 7:**
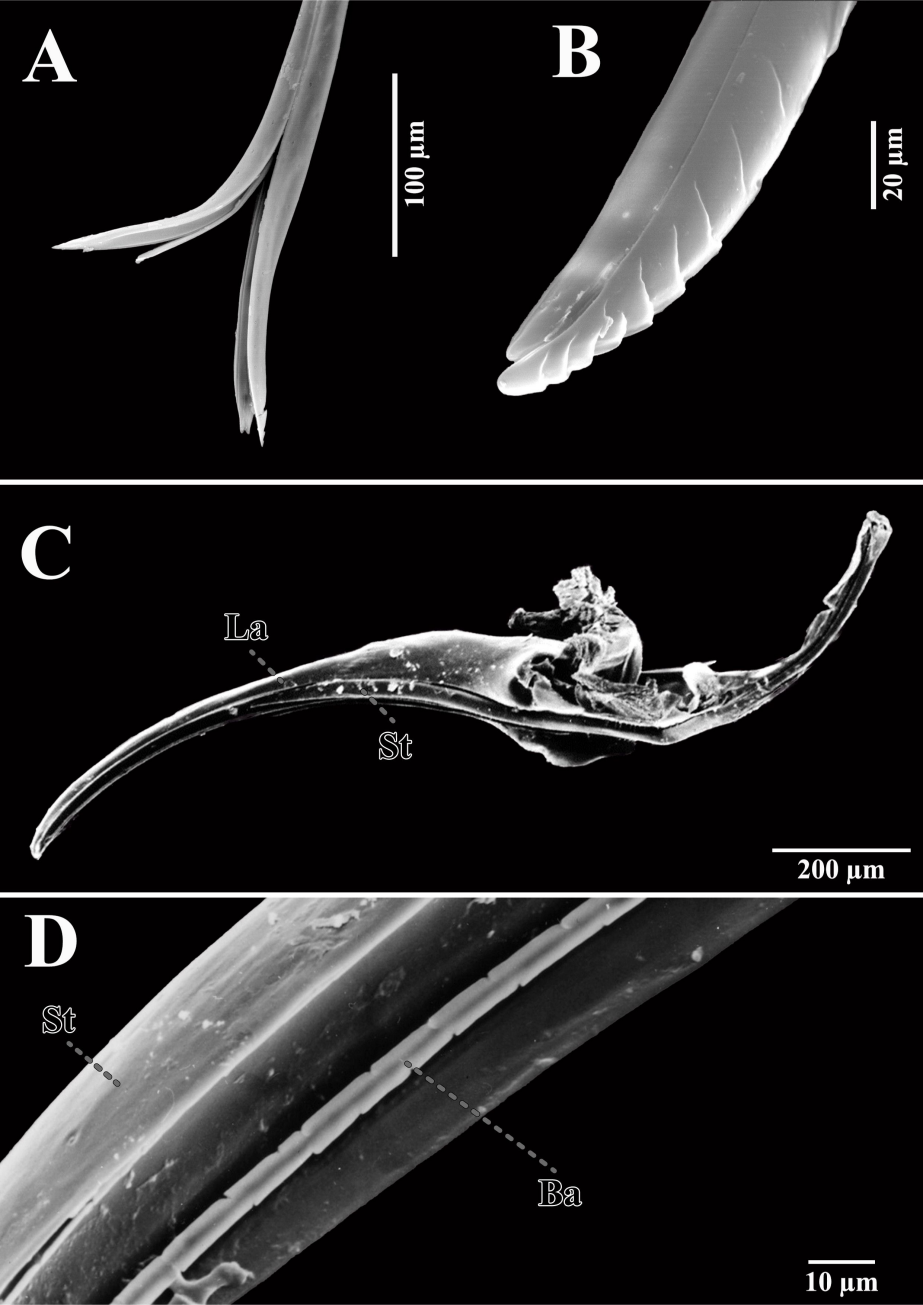
Perforating structures on the metasoma of Hymenoptera. (A, B) Ovipositor of *Neralsia* sp. and *Andricus coriarius*. (C, D) Sting of *Oxybelus haemorrhoidalis*. La: lancet; St: stylet; Ba: lateral barbed structure.

**Sting and ovipositor – drills, probes and needles:** Parasitoidism is a specialized form of carnivory wherein the parasitoid completes its entire life cycle by feeding exclusively on a single host individual. This strategic adaptation is prevalent among Hymenoptera, with an estimated 70% of known hymenopteran species embracing this lifestyle [153]. As a result of evolutionary processes, hymenopterans have been bestowed with a highly specialized organ used for laying eggs, the ovipositor. The hymenopteran ovipositor consists of two pairs of valvifers located at the base, which house the muscles controlling the ovipositor mechanism, along with three pairs of valvulae capable of sliding along each other [154] (Figure 7A,B). The size of this structure can vary significantly, ranging from micrometers to the longest ovipositors documented in Arthropoda with lengths of over 100 mm [155–156], facilitating oviposition in diverse substrates such as wood, soil, or within other organisms [157] (Figure 1F, Figure 7B).

The ovipositor of certain parasitic wasps, such as those in sawflies wasp and ichneumon wasps has inspired the development of a prototype of a drill rasp designed for excavating femoral cavities, tailored for snugly inserting cementless hip prosthesis stems [158]. Apart from their highly specialized form, also the elemental structure of this organ evolved to increase drilling ability while limiting abrasion and wear. Indeed, trace metals such as Zn, Mn, and Cu were variably found within the cuticle matrix of Hymenoptera ovipositors and stings [159–160]. The mechanics of the ovipositor, characterized by its flexibility, strength, and the ability to penetrate tough materials with minimal force, offer a model for designing minimally invasive surgical instruments and precision technology [158]. Still in the medical field, the ovipositor’s unique properties may lead to the development of highly specialized probes and needles for diagnostic and therapeutic purposes [161–162]. These bioinspired tools can navigate through soft tissues with minimal damage, mimicking the ovipositor’s ability to penetrate substrates smoothly and efficiently [158,161–163]. Hence, drawing inspiration from ovipositors, these innovations may enhance medical procedures by amplifying precision, minimizing recovery periods, and augmenting patient outcomes, particularly in scenarios where maneuverability and accuracy are paramount.

In addition to medical applications, the ovipositor’s design principles have broader implications for engineering and technology. The ovipositor’s ability to penetrate tough materials with minimal force inspires the development of tools and machinery that require high precision and efficiency [164]. For instance, researchers at the Surrey Space Centre have drawn inspiration from the wood wasp to create a drilling system that is simple, robust, lightweight, and efficient for use on extra-terrestrial subsurface exploration [165–168]. The ovipositor’s structural and functional characteristics can inform the design of robotic systems. Robots equipped with ovipositor-inspired appendages can perform delicate tasks that require both strength and precision, such as handling fragile objects or performing intricate assembly operations [168]. Therefore, drilling and cutting tools modeled after the ovipositor can achieve greater accuracy and reduce wear and tear, leading to longer lasting and more effective equipment. This innovation can be applied in various industries, from construction to manufacturing, enhancing productivity, and reducing operational costs.

In environmental science, the study of ovipositor mechanics can contribute to the development of sustainable technologies for soil sampling and ecological monitoring. Devices modeled after the ovipositor can penetrate the ground to collect soil samples without causing significant disturbance to the ecosystem. This capability is important for monitoring soil health, studying underground biodiversity, and assessing environmental impacts, providing researchers with valuable data while preserving natural habitats.

**Delivery systems:** Strength and efficiency of the ovipositor also offer insights into creating advanced drug delivery systems [169]. These systems can deliver medications directly to targeted areas within the body, improving the efficacy of treatments while minimizing side effects [170–171]. By emulating the ovipositor’s ability to penetrate and deliver substances precisely, researchers have developed microneedles [163]. This approach is especially beneficial for administering vaccines and treatments for chronic conditions, providing a less invasive and more patient-friendly alternative to traditional methods.

The Aculeata sting represents a remarkable evolutionary transformation of the ovipositor. It has lost its original function of egg-laying to serve as a potent venom delivery system (Figure 7C,D). The venom delivery systems of Hymenoptera are precise and efficient, inspiring the design of microinjection systems and targeted drug delivery methods that minimize collateral damage to surrounding tissues [170]. Drawing inspiration from the precision and efficiency of Hymenopteran venom delivery systems, advanced microinjection devices can be crafted for laboratory applications, boasting painless insertion and extraction, minimal dermal injuries, mechanical durability, and suitable biocompatibility [172]. Furthermore, the capacity to induce mechanical tissue damage may vary across species, with those species employing the sting for offensive or defensive purposes potentially possessing cuticular microstructures proficient at inflicting significant harm [173].

**Figure 8:**
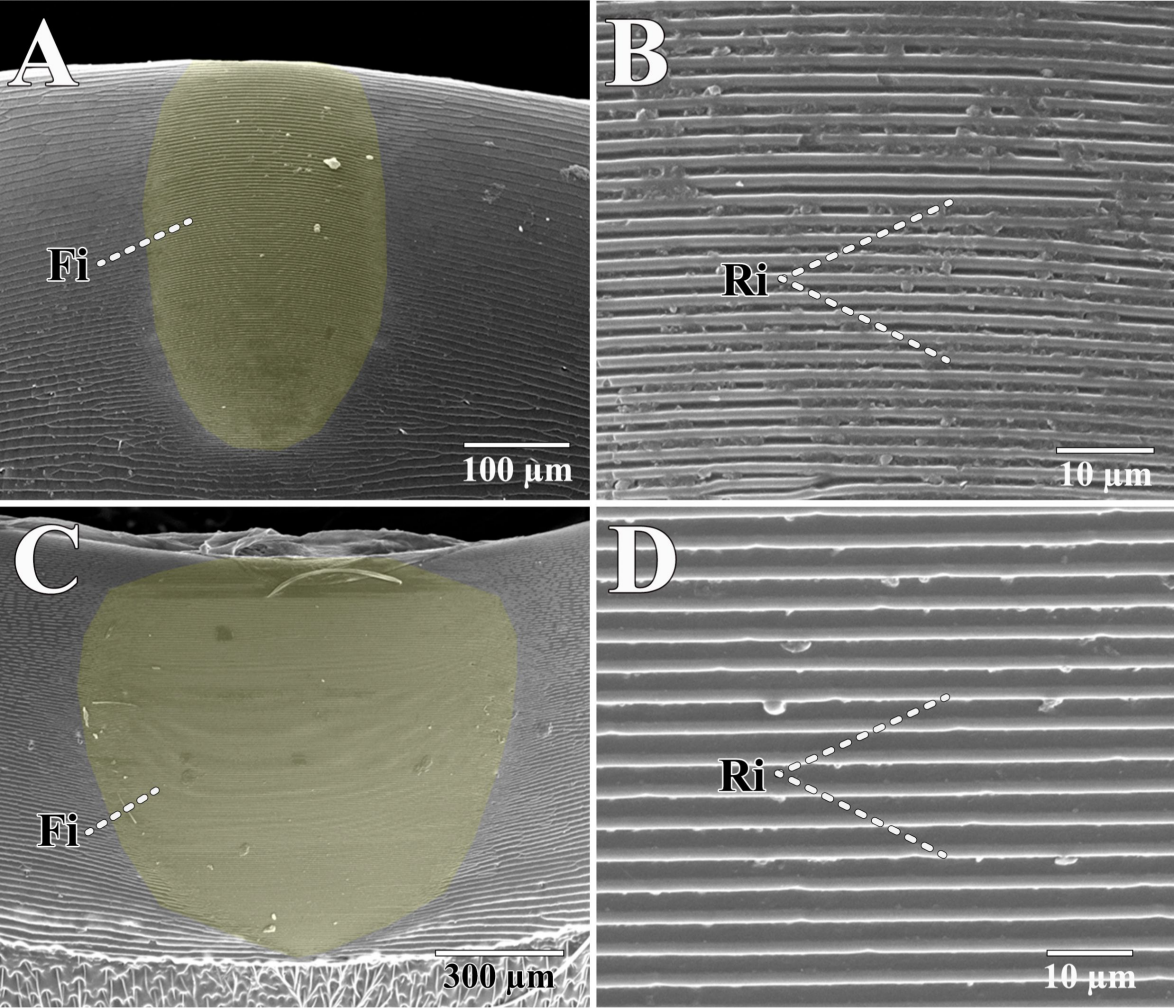
Scanning Electron Microscopy image showing the stridulatory organ in male velvet ants. (A, B). *Myrmilla capitata*; (C, D). *Nemka viduata.* Fi: file; Ri: ridges. Sound is produced by rubbing files and ridges against each other.

**Stridulatory organ:** Stridulation in Hymenoptera, the process by which these insects produce sound by rubbing certain body parts together [174], primarily involves the interaction between two tergites of the metasoma [174] (Figure 8). This natural mechanism, involving specialized structures such as ridges and scrapers, can generate a wide range of frequencies with precision and efficiency, providing valuable insights for the advancement of sophisticated acoustic and vibration technologies. In nature, this acoustic strategy is likely employed for alarm signaling, sexual behavior, and as a versatile communication channel among eusocial insects (i.e., ants) [175–178]. By studying and mimicking these biological systems, it is possible to create innovative solutions in various fields, including communication, medical devices, and materials science.

Certain hymenopterans are capable of emitting stridulatory sounds at high frequencies, including ultrasound (≥20 kHz) [179–180]. The design of medical devices could greatly benefit from innovations inspired by these stridulation mechanisms. Ultrasound technology has demonstrated significant potential in diagnostic imaging and ultrasound-responsive drug delivery [181–182]. Ultrasound technology is particularly promising for cancer treatment and disease modulation, as it facilitates the delivery of therapeutic agents such as genetic material, proteins, and chemotherapeutics [182–183]. Incorporating principles from the finely tuned frequency production observed in Hymenoptera could significantly enhance this technology, resulting in more precise and effective ultrasound equipment, thus improving patient outcomes in both diagnostics and treatment. Furthermore, studying the use of acoustic signals in hymenopterans may offer valuable inspiration for future research, as these context-dependent signals can modulate the production or inhibition of chemical signals in other individuals [178].

Moreover, the precise control over vibration frequencies observed in stridulation can inspire advancements in industrial applications where vibration control is essential. This includes machinery that operates more quietly and efficiently, reducing noise pollution and wear on components. Additionally, the principles of stridulation could be applied to develop sensors and actuators that are more sensitive and responsive, improving the performance of various mechanical and electronic systems.

**Metasomal shape:** The morphology of the metasoma in Hymenoptera offers significant potential for biomimetic applications, particularly within aerospace engineering. For instance, the segmented and flexible structure of a bee’s metasoma facilitates efficient and dynamic flight maneuvers [184]. Bees optimize their aerodynamic performance and maneuverability through biomorphic adjustments to their shape [185]. This adaptable structure can inspire the development of aerospace vehicles with enhanced axial scalability and bending properties [185]. Incorporating a morphing structure inspired by the Hymenoptera metasoma could lead to the creation of supermaneuverable flight systems, contributing to advanced designs for aircraft and unmanned aerial vehicles, and reducing aerodynamic drag [185].

Furthermore, the metasoma of bees can dissipate residual flight energy during landing [186], and the shape of the metasoma can influence maneuverability and flight speed in wasps. Integrating these biological principles could result in vehicles capable of agile and precise movements and increased stability in turbulent conditions. Despite these promising possibilities, the shape and surface characteristics of the hymenopteran metasoma remain underexplored, and families with unique metasomal forms still lack basic research. For instance, cuckoo wasps (Chrysididae) possess a concave metasomal venter, enabling them to roll into a ball when threatened [187]. Hatchet wasps (Evaniidae) exhibit short and flag-shaped metasoma that moves up and down as they walk, resembling a flag or hatchet. In pelecinid wasps (Pelecinidae), the elongated metasoma allows them to locate and deposit eggs in subterranean hosts [188]. This unique metasomal morphology provides various adaptive advantages and can inspire innovations in designs.

**Prey-carrying and prey-catching mechanisms:** Some groups of predatory wasps hunt prey and subsequently transport it in flight to the nests, using either mandibles, legs or even the sting to keep the prey. Most predatory wasps use front and mid legs to carry the prey, while a few wasp species hold their prey only with the hind legs [189]. The efficient prey-loading mechanisms of some of these Hymenopteran species, that can even carry lift loads greater than the theoretical maximum as expected by their body mass and wing morphology [190], can inspire the design of robotic systems for material handling and transport, enhancing the efficiency of automated loading and unloading processes in various industries. By holding the prey on the sting, some species of *Oxybelus* (Crabronidae) have effectively freed all three pairs of legs for other purposes [189]. This peculiar way to carry the prey impaled on the aculeus is made possible through the special curvature and surface sculpture of sting elements (notably, barbs on the distal part of the lateral surface of the first valvula) [191–192], as well as by modifying the wings lift coefficient between the anterior and posterior wings movement [193].

### Other features

#### Interlocking structures

Hymenoptera exhibit interlocking structures in their bodies that provide stability and flexibility. These structures can inform the design of modular and reconfigurable systems in robotics and materials science, especially for drones that must carry attachable items. Phoretic copulation is probably the main inspiration source [194]. Similar interlocking mechanisms can be found between male mouthparts and female head and mesosome for phoretic copulation (e.g., some mutillids) [195], and the same happens for male–female genitalia (e.g., bethylids, thynnids) [196]. Another example is the labrum–maxillae interlocking mechanism exclusive of ants [197].

#### Acarinaria

Mites can be found in various locations on the bodies of hymenopterans [198]. However, some species of bees and wasps have evolved specialized structures known as acarinaria, which facilitate the safe transport of mites [198–200]. The acarinarium can be located in one or more areas on the insect’s body surface such as the propodeum, mesosoma, just below the apex of the first metasomal tergum, and in some cases, within the genital chamber of species such as *Ancistrocerus antilope* (Vespidae) [200]. These structures could be integrated into drones, plant leaves, or stems, providing secure havens for biocontrol agents. This approach would naturally manage pest populations, reducing the need for chemical pesticides and promoting sustainable farming practices [201–203].

## Conclusion

The diverse and highly specialized features of Hymenoptera offer a treasure trove of inspiration for biomimetic applications. By studying these insects, researchers can develop innovative materials and devices that mimic their efficiency and functionality, leading to advancements in medical technology, robotics, environmental monitoring, and beyond. The ongoing exploration of Hymenopteran biology and mechanics continues to unlock new possibilities for technological innovation, underscoring the profound impact of nature-inspired designs.

## Data Availability

Data sharing is not applicable as no new data was generated or analyzed in this study.
